# Investigating the link between medical urgency and hospital efficiency – Insights from the German hospital market

**DOI:** 10.1007/s10729-020-09520-6

**Published:** 2020-09-16

**Authors:** Annika Maren Schneider, Eva-Maria Oppel, Jonas Schreyögg

**Affiliations:** Hamburg Center for Health Economics, University of Hamburg, Esplanade 36, 20354 Hamburg, Germany

**Keywords:** Urgency, Technical efficiency, Hospitals, Data envelopment analysis, Double-bootstrap

## Abstract

With hospital budgets remaining tight and healthcare expenditure rising due to demographic change and advances in technology, hospitals continue to face calls to contain costs and allocate their resources more efficiently. In this context, efficiency has emerged as an increasingly important way for hospitals to withstand competitive pressures in the hospital market. Doing so, however, can be challenging given unpredictable fluctuations in demand, a prime example of which are emergencies, i.e. urgent medical cases. The link between medical urgency and hospitals’ efficiency, however, has been neglected in the literature to date. This study therefore aims to investigate the relationship between hospitals’ urgency characteristics and their efficiency. Our analyses are based on 4094 observations from 1428 hospitals throughout Germany for the years 2015, 2016, and 2017. We calculate an average urgency score for each hospital based on all cases treated in that hospital per year and also investigate the within-hospital dispersion of medical urgency. To analyze the association of these urgency measures with hospitals’ efficiency we use a two-stage double bootstrap data envelopment analysis approach with truncated regression. We find a negative relationship between the urgency score and hospital efficiency. When testing for non-linear effects, the results reveal a u-shaped association, indicating that having either a high or low overall urgency score is beneficial in terms of efficiency. Finally, our results reveal that higher within-hospital urgency dispersion is negatively related to efficiency.

**Electronic supplementary material:**

The online version of this article (10.1007/s10729-020-09520-6) contains supplementary material, which is available to authorized users.

## Highlights

- This study provides novel insights into how hospitals’ urgency characteristics influence their efficiency.
- We calculate two innovative hospital-level measures that reflect the medical urgency of cases treated by a hospital per year: (1) urgency score and (2) urgency dispersion.
- Our results indicate that with the medical urgency score increasing, hospitals’ efficiency is decreasing. Furthermore, we find non-linear effects for this relationship.
- Looking at the dispersion of medical urgency within a hospital, we see that efficiency losses are particularly detrimental in hospitals treating both low and high urgency cases.
- Policy makers, researchers and practitioners are well-advised to acknowledge the influence of medical urgency in assessing hospital performance. Focusing on a homogeneous case composition with regard to medical urgency might be a means for hospitals to avoid efficiency losses or even increase their efficiency.

## Introduction

Enhancing efficiency has become an increasingly important way for hospitals to deal with the growing competitive pressures in hospital markets. At the same time, unpredictable fluctuations in demand can make it challenging for hospitals to operate efficiently [1, 2]. Such fluctuations are driven in particular by a disproportionate increase in emergency cases in many healthcare systems. In Germany, for example, the number of emergency cases rose by 46% between 2005 and 2013, while the number of elective cases increased by only 1.4% [3]. Similar patterns can be observed in other countries, such as Australia, the United Kingdom, and the United States [4, 5]. In contrast to elective cases, which can usually be pre-arranged and are therefore highly predictable, demand for emergency cases is less predictable [3, 6]. In addition, emergency cases usually comprise patients with highly urgent conditions that require immediate treatment. Indeed, the overall medical urgency of hospital cases might be crucial when it comes to providing hospital services in an efficient manner. Since the composition of cases differs between hospitals, i.e. some hospitals treat relatively more urgent cases than others, the question whether urgency characteristics of hospitals’ case composition affect hospitals’ efficiency seems to be very pressing.

In characterizing the composition of hospital cases, researchers frequently focus on the overall severity of hospital cases, which is most frequently captured by case mix or case mix index measures [7]. In this study, we extend this research by focusing on the overall medical urgency of hospital cases as further essential characteristic that describes hospitals’ case composition.

Referring to production theory, we propose that by capturing the hospitals’ medical urgency characteristics, research may better explain variation in efficiency at the hospital level. Surprisingly, despite extensive literature on hospital efficiency and its determinants [8], the link between efficiency and urgency characteristics of hospitals has not been investigated to date. While initial evidence points to the importance of emergency care in explaining hospital productivity and efficiency [9–11], this research has only a very limited relevance in explaining how the urgency characteristics affect their efficiency. However, prior research on the (performance) implications of emergency care in hospitals offers valuable guidance in developing our reasoning on potential mechanisms through which hospitals’ urgency characteristics might affect their efficiency.

High urgency medical treatment may lead to fragmentation of production processes. Indeed, observational evidence suggests that physicians in hospital specialties that deal with many urgent cases, such as emergency physicians, often report high numbers of work interruptions [12, 13], which might cause inefficiencies. In addition, high urgency cases must often be prioritized over low urgency ones when it comes to allocating scant or occupied resources, such as personnel or operating room capacity. In the event of capacity constraints, short-notice prioritizations might lead to inefficiencies due to canceled or postponed non-urgent surgeries [14]. This, in combination with bed competition from emergency admissions, has been shown to result in longer hospital stays or inpatient waiting times for elective cases [4]. Moreover, differences in the predictability of elective and medically urgent cases might be pivotal in the context of efficiency. More specifically, the unpredictability of medical urgencies makes it difficult to optimize the planning and use of resources [6]. Indeed, prior research has shown that hospitals respond to demand uncertainty by changing their cost structure and production [1, 15], which might, in turn, also lead to inefficiencies because more standby capacity needs to be maintained causing a higher risk that this capacity will remain unused [16].

The aim of the present study is to build on and extend prior efficiency research by linking hospitals’ urgency characteristics to their technical efficiency. To do so, we use a large data set from hospitals in Germany comprising 4094 hospital observations for 2015, 2016, and 2017. For this study, we propose two novel measures to describe hospitals’ urgency characteristics. The hospital’s urgency score (UrS) describes the average level of medical urgency of all cases treated in a hospital. Some hospitals might have a composition of cases in which elective care predominates (low UrS), while others have a composition of cases in which emergency care predominates (high UrS). Using this measure, conclusions can be drawn about whether hospitals with a lower UrS are more or less efficient than hospitals with a higher UrS. We also test for non-linear relationships in the UrS-efficiency link. In addition to the average urgency level captured by the UrS, we investigate within-hospital dispersion of urgency (UrD). We argue that the UrD is also relevant for analyzing efficiency, since it captures the urgency diversity in the hospitals’ case composition. We exemplify our UrD reasoning using a simplified scenario with two hospitals (A and B) both treating two patients. Hospital A’s patients both suffer from conditions with medium urgency. Hospital B treats one patient with low urgency and one patient with high urgency. The resulting hospital-level UrS for both hospitals would be in a medium range. However, hospital A’s cases are homogeneous regarding their urgency, whereas hospital B’s patients are rather divers. Hence, low UrD indicates that the composition of cases in a hospital is rather homogeneous, whereas high UrD indicates that it is rather diverse. Our assumption that UrD might relate to hospitals’ efficiency is rooted in the hospital specialization literature, which indicates that focusing on homogeneous groups of diagnoses and procedures is associated with increased efficiency [17, 18]. Further information on the calculation of our two novel hospital-level urgency measures are presented in the methods section (2.4.1).

We use the two-stage data envelopment analysis (DEA) approach with double bootstrap and truncated regression analysis proposed by Simar and Wilson [19] in order to estimate bias-corrected technical efficiency scores and obtain valid inferences about the relationships in question. Hospitals are assumed to be technically efficient if they produce a given amount of output with the minimal amount of input (input-orientation) [20]. Further information on the methodological approach applied in this study is presented in chapter 2.2.

The main innovation of our study is the detailed analysis of the link between hospitals’ urgency characteristics and their efficiency by investigating not only the average level of urgency of cases treated in a hospital but also the dispersion of cases with different levels of urgency. Our findings provide important insights for hospital managers and policy makers who are seeking effective ways to enhance hospital efficiency.

## Methodology

Table 1 provides a comprehensive outline of our methodological approach. The data selection procedure and individual steps in our analyses are described in detail in the following sections.

**Table 1:** Overview of methodological approach

Step	Description
1. Collecting data from two sources *(subsection “Data sets”).*	*a) Hospital data from the annual mandatory quality reports published by almost all hospitals*^*a*^ *in Germany* - Data from 2365 German acute care hospitals (hospital-site level) were extracted for the years 2015, 2016, and 2017 (unbalanced panel). - Contains hospital-level information on inputs (beds and hospital staff), outputs (inpatient and outpatients cases), ICD-10 main diagnoses, and hospital characteristics (e.g., hospital ownership and teaching status). *b) Data from the Federal Institute for Research on Building, Urban Affairs, and Spatial Development (BBSR)* - Data from 401 German districts were extracted for the years 2015, 2016, and 2017 - Contains information on type of region in which each hospital is located
2. Merging data, defining exclusion criteria, and checking plausibility of data *(subsection “Data sets”).*	To ensure the comparability of the hospitals in the sample, we excluded - hospitals with fewer than 50 beds - university hospitals - hospitals providing only psychiatric, pediatric, or geriatric care - rehabilitation centers - day and night clinics Additional plausibility checks (completeness and correctness of the data) were performed. A total of 1428 hospitals and 4094 hospital year observations remained in the sample.
3. Selecting inputs, outputs, and independent variables for the second-stage regression analysis *(subsections “Inputs and output specification” and “Contextual variables of the second stage”.*	*Inputs*: - hospitals’ medical staff in fulltime equivalents (FTE): registered nurses, assistant nurses, and physicians - hospital beds *Outputs*: - adjusted inpatient cases - outpatient cases *Contextual variables of the second stage:*
4. Applying double bootstrap data envelopment analysis (DEA) and running truncated regression analyses *(subsection “Operational model”).*	- Main variables of interest: hospitals’ urgency score (UrS) and within-hospital urgency dispersion (UrD) were calculated based on medical urgency values proposed by Krämer et al. [3] - Control variables: hospital ownership, academic teaching status, Herfindahl-Hirschman index (HHI) as a proxy for hospital competition, type of region in which hospitals were located, and year dummies.
	Application of an input-oriented variable returns to scale model for all hospitals in the dataset (intertemporal frontier). Deriving bias-corrected DEA efficiency scores and obtaining valid inferences on the second-stage contextual variables using bootstrapped truncated linear regression following algorithm #2 as proposed by Simar and Wilson [19].

^a^ Since 2005, German hospitals have been legally obliged to publish quality reports, in which they have to provide, for instance, information about their organizational structures, staffing, case numbers, as well as provided services and treatments. This affects all hospitals in Germany that are authorized to bill German sickness funds for inpatient services

### Data sets

We combined data from two different sources: First, we used the mandatory, structured quality reports published by German hospitals for the years 2015, 2016, and 2017. This extensive data set covers all hospitals in Germany at the level of individual hospital sites (*n* = 2365). From this data set, we obtained information on inputs, outputs, and hospital characteristics. To ensure the comparability of production processes, we excluded hospitals with fewer than 50 beds, university hospitals, psychiatric hospitals, rehabilitation clinics, day and night clinics, and hospitals specialized in pediatric or geriatric care [21, 22]. To identify outlying observations due to data errors, we conducted systematic plausibility checks. Second, we drew upon administrative data from the Federal Institute for Research on Building, Urban Affairs, and Spatial Development (BBSR) to obtain information on the location of the hospitals. We merged hospital-level data for the years 2015 through 2017 with administrative data for the same years. Ultimately, the unbalanced final sample consisted of 4094 observations from 1428 acute care hospitals. In line with previous efficiency studies, we estimated an intertemporal frontier [21–23]. In doing so, we merged the data for all years into one dataset.

### Operational model

To estimate hospital efficiency, we used the DEA approach introduced by Charnes, Cooper, and Rhodes in 1978. DEA is a nonparametric modelling technique to estimate a best-practice production frontier based on observed data points and to assess the relative efficiency of decision making units (DMUs) against this frontier [24, 25]. One advantage of DEA is its ability to accommodate multiple outputs and inputs simultaneously, which would appear to be of particular significance when investigating the efficiency of service organizations, such as hospitals that have a complex production technology [21]. Furthermore, DEA does not require an a priori assumption about the functional form of the production frontier (i.e., about how the inputs are transformed into outputs). Indeed, DEA is deterministic in nature, i.e., it uses linear programming to construct a frontier based on the values that are observed in the sample. In the process, it is implicitly assumed that all observations belong to the production set and inefficiency is measured as the (radial) distance of the DMU to the best practice frontier – that is, no allowance is made for statistical noise. Like other nonparametric estimators, DEA has a slow rate of convergence that becomes worse with an increasing number of inputs and outputs relative to the number of observations in the sample [26]. Although our sample size might be sufficiently large, we checked whether our estimations could be affected by the ‘curse of dimensionality’ based on the diagnostics proposed by Wilson [26]. The obtained diagnostics relating to the effective sample size as well as the proportion of DMUs with efficiency estimates of 1 in a Free Disposal Hull (FDH) model indicated that the curse of dimensionality might not affect our estimates.^1^

For our study, we calculate hospitals’ technical efficiency. In general, hospitals can be assumed to be technically efficient if they produce a given amount of output with the minimal amount of input (input-orientation) or if they maximize output given a fixed amount of input (output-orientation) [20]. In this study, following prior hospital efficiency research [8], we use an input-orientated DEA model. Assuming that hospitals have greater control over their inputs (e.g., over their staff) rather than their outputs (e.g., inpatient cases), an input-oriented DEA model seems to be more appropriate than an output-orientated DEA model. Furthermore, we allowed for variable returns to scale (VRS).^2^ The technical details of the underlying linear programming problem of the Farrel input-orientated technical efficiency model under VRS can be found in the electronic supplementary material (ESM 1) and are also comprehensively described elsewhere [23, 28, 29].

When investigating factors that might influence hospital efficiency, a widely used approach is the two-stage analysis, in which efficiency scores are estimated in the first stage using DEA and these estimates are subsequently used as dependent variables in a second-stage regression analysis (see ESM 2). Because the true efficiency score is unknown and must therefore be approximated using estimated DEA efficiency scores, the conventional two-stage analysis, applying for example (censored) Tobit or ordinary least squares (OLS) regression in the second stage, has been shown to fail in obtaining valid inferences [19]. Particularly, Simar and Wilson stressed that the DEA efficiency scores obtained in conventional two-stage approaches are biased and serially correlated by construction. To overcome these limitations, we lined up with recent research on applied efficiency analysis in healthcare organizations [8, 23, 30, 31] and followed the algorithm #2 steps described by Simar and Wilson [19], in which a two-stage DEA analysis with truncated regression and a double bootstrap procedure is recommended. This approach enabled us to obtain valid inferences in two-stage efficiency models while producing standard errors and confidence intervals for both efficiency estimates and coefficients at the same time. A comprehensive overview on the individual steps proposed in algorithm #2 is presented in the electronic supplementary material (ESM 3). To estimate bias-corrected efficiency scores and construct estimates of confidence intervals, one must choose a sufficiently large number of bootstrap replications, *L*_1_ and *L*_2_, respectively. For our analyses, we used *L*_1_= 100 and *L*_2_ = 2000, which are the number of replications proposed by Simar and Wilson [19]. We undertook all analyses using the *simarwilson* package implemented in Stata Version 15 (StataCorp LP, College Station, TX).

One central assumption in two stage approaches is the ‘separability condition’, which means that the environmental factors used in the second stage as independent variables explain deviations from the efficient frontier but do not influence the technology frontier itself. In line with recent research investigating variations in health care organizations’ efficiency, we continued with the analysis assuming that the assumption of ‘separability’ holds and acknowledge that our further analyses mainly rely on heuristic assessment of the production process [30–33].

### Input and output specification

When selecting inputs and outputs, we focused on inputs and outputs that were theoretically meaningful and have been linked consistently to the technical efficiency of hospitals [8, 34]. To ensure comparability with previous studies applying the double bootstrap approach [23, 30], we propose a radial rather than a non-radial DEA model.^3^

In our main specification, we included a set of four inputs and two outputs to describe hospitals’ production technology. As inputs, we included hospital staff measured in fulltime equivalents (FTE) – i.e. registered nurses, assistant nurses, and physicians – as well as inpatient beds. The breakdown of FTEs into several FTE groups is in line with related research in this field [23, 37]. Nurses and physicians are the bedside staff who are involved most directly in providing patient care and are therefore considered to be the most significant input factor in the process of healthcare delivery [38]. The significance of these occupational groups is also reflected in the share of total operating expenditure accounted for by their personnel costs, namely on average 63% in German hospitals [39]. Furthermore, we included hospital beds as an input. Given that in Germany investment costs are allocated based on the number of beds (lump sums per bed), the number of hospital beds is a suitable proxy for capital input and has also been used in the international hospital efficiency literature [8, 21, 23, 30, 40].

We used two hospital outputs: (1) inpatient cases and (2) outpatient cases, which constitute the most important outputs for general care hospitals [34] and are used frequently in hospital efficiency studies [8]. Regarding inpatient cases, it is important to adjust for case severity because not all patients need the same level of treatment and attention^4^ [40]. Following prior research [41–43], we used the case mix adjustment based on the relative length of stay (LOS) for groups of hospital diagnoses as proposed by Herr [44]. We grouped hospital diagnoses according to the German ICD-10 catalogue into a total of *G* = 241 diagnosis groups. Information on the average length of stay in each diagnosis group, *LOS*_*g*_, was extracted from the hospital statistics published by the German Federal Statistical Office [45]. The weights *π*_*g*_ for each diagnosis group *g* were then calculated based on the following formula:

1 $$ {\pi}_g=\frac{LOS_g}{\frac{1}{G}\ {\sum}_{g=1}^G{LOS}_g};g=1,\dots, G. $$

For each hospital *j*, we then multiplied the number of inpatient cases (*inpatients*) in a diagnosis group with its respective weight to obtain the total adjusted number of hospital cases:

2 $$ inpatients\_{adj}_j={\sum}_{g=1}^G{\pi}_g\bullet {inpatients}_{g,j};\mathrm{g}=1,\dots, G. $$

### Contextual variables in the second stage

#### Medical urgency

For this study, we calculated two hospital-level variables describing hospitals’ urgency characteristics: the hospitals’ urgency score (UrS) and the within-hospital urgency dispersion (UrD). To calculate these measures we used the medical urgency values proposed by Krämer et al. [3]. The authors used supervised machine learning methods (random forest) to develop a classification scheme that assigns urgency values (*u*) between 0 and 1 to each relevant primary diagnosis in the ICD-10-GM catalogue.^5^ The random forest estimation provided individual class probabilities for each ICD diagnosis to belong to either emergency or elective care. These probabilities serve as a measure of medical urgency. A high urgency value for a diagnosis *d* is indicative for high medical urgency. Krämer et al. [3] classified diagnoses with an urgency value below 0.5 as elective and diagnoses with an urgency value above 0.5 as emergency.

Since our unit of analysis is the hospital rather than the individual patient, we calculated the hospitals’ UrS as the average score across cases treated of each hospital *j*:

3 $$ {UrS}_j=\frac{\sum_{d=1}^D{u}_d\bullet {inpatients}_{d,j}}{\sum_{d=1}^D{inpatients}_{d,j}}, $$

with *u* being the urgency value for ICD diagnosis *d* at the 4-digit level and *inpatients* being the number of inpatient cases with this diagnosis. As a result, we received a value between 0 and 1. Adapting the interpretation of the urgency values proposed by Krämer et al. [3], the hospital-level UrS can be interpreted as follows: Hospitals with an overall UrS below 0.5 have a case composition in which elective care predominates, and hospitals with an overall UrS equal or above 0.5 have a case composition in which emergency care predominates.^6^ In our analyses, we included the UrS of a hospital as both, a linear term (Model I) and as a squared term (Model II) to test for non-linearity, i.e. to investigate whether the UrS had non-linear effects.

To capture the degree of urgency diversity in hospitals’ case composition, we calculated the standard deviation of each hospital’s UrS, which we define as within-hospital urgency dispersion (UrD):

4 $$ {UrD}_j=\sqrt{\frac{\sum_{d=1}^D{inpatients}_{d,j}\bullet {\left({u}_d-{UrS}_j\right)}^2}{\left({\sum}_{d=1}^D{inpatients}_{d,j}\right)-1}} $$

The parameters are defined as above in Eq. (3). A value close to zero (low UrD) indicates that a hospital has a case composition that is homogeneous in terms of medical urgency, whereas larger values (high UrD) indicate that a hospital has a case composition that is diverse in terms of medical urgency. Hence, UrD is referring to levels of urgency of individual cases and how these are spread out across the composition of cases of a given hospital *j*, e.g., lots of very urgent cases, lots of cases with medium urgency, or an equal spread from low to high urgency.

#### Control variables

In our second-stage truncated regression models, we included several control variables which have been shown to explain variation in hospitals’ efficiency.

First, we controlled for hospital ownership using a set of dummy variables, i.e. public, private nonprofit, and private for-profit, with public hospitals as the reference group. Hospital ownership type has been found to affect hospital efficiency [17, 41, 46, 47]. Referring to public choice [48] or principal agent theory [49], it has been assumed that public hospitals have a higher goal plurality and poorer control mechanisms, both of which can reduce incentives to enhance efficiency. Private hospitals, in contrast, are assumed to act in a market-oriented fashion, with their goals supporting efficient behavior. Second, we used a binary variable to account for hospitals’ academic teaching status, with a value of 1 representing teaching hospitals. It is generally assumed that academic teaching might limit the productivity of medical work, which could lead to efficiency losses [50].

We used the Herfindahl-Hirschman Index (HHI) as a measure of market concentration within a hospital’s unique market area. The HHI is commonly used as a proxy for competitive pressures in a hospital’s market and has been shown to be related to hospital efficiency [23, 51]. We calculated the HHI based on inpatient discharges and defined the area within a 32 km radius of a hospital as its catchment area [41]. To take into account that hospitals do not compete in all medical disciplines, we calculated a separate HHI for each of the 22 ICD-10-GM chapters and calculated an average HHI, weighting each chapter-specific HHI by the proportion of patients a hospital treated in that chapter. The HHI is scaled to an interval ranging from 0 to 1, with 0 indicating the highest level of competition. We also controlled for hospitals’ location [32, 41] using a set of dummy variables, i.e. large cities, urban district, rural district, and a remote district, with large cities as the reference group. Finally, we included year dummies (2015, 2016, and 2017 with 2017 as the reference group) to capture potential trend effects between observational years. A detailed description of study variables is provided in Table 2.

**Table 2:** Summary of study variables

Variable	Description	Mean/freq	SD
Input*s*			
Beds	Number of acute medical beds in a hospital by the reporting date of 31 December	279.43	210.38
Physicians	Annual average number of FTE physicians	84.71	81.54
Registered Nurses	Annual average number of FTE registered nurses (three years of apprenticeship), including the following professions: nurses, midwifes, surgical assistants, and medical assistants	209.52	184.68
Nurse assistants	Annual average number of FTE nurse assistants, including all nursing professions with fewer than three years of apprenticeship	12.17	15.03
Outputs			
Adjusted inpatient discharges	Number of (weighted) inpatient discharges: case mix adjustment based on the relative LOS in different diagnostic categories	10,687.06	8607.35
Outpatient cases	Number of outpatient visits (hospitals count each outpatient contact by a patient with the organizational units)	21,778.93	26,137.96
Contextual factors			
UrS	Average level of urgency across all main diagnoses of patients treated in the hospital in the reporting year	0.44	0.13
UrD	Standard deviation of the individual hospitals’ UrS	0.31	0.05
Ownership	Public (reference group)	0.34	
	Private nonprofit	0.44	
	Private for-profit	0.22	
Teaching status	Hospitals’ involvement in academic teaching, binary variable	0.57	
Competition	Herfindahl-Hirschman-Index (HHI), 32 km fixed radius, with 0 indicating less concentrated markets (high competition) and indicating 1 highly concentrated markets (low competition).	0.16	0.12
Location	Large cities: cities with more than 100,000 inhabitants (reference group)	0.28	
	Urban district: districts with a population density of more than 300 inhabitants per km^2^	0.35	
	Rural district: districts with a population density of more than 150 inhabitants per km^2^.	0.18	
	Remote district: districts with a population density of less than 150 inhabitants per km^2^	0.19	

Pooled dataset with *n* = 4094; *FTE* fulltime equivalent, *LOS* length of stay, *UrD* within-hospital urgency dispersion, *UrS* urgency score

## Results

### Descriptive statistics

**Fig. 1:**
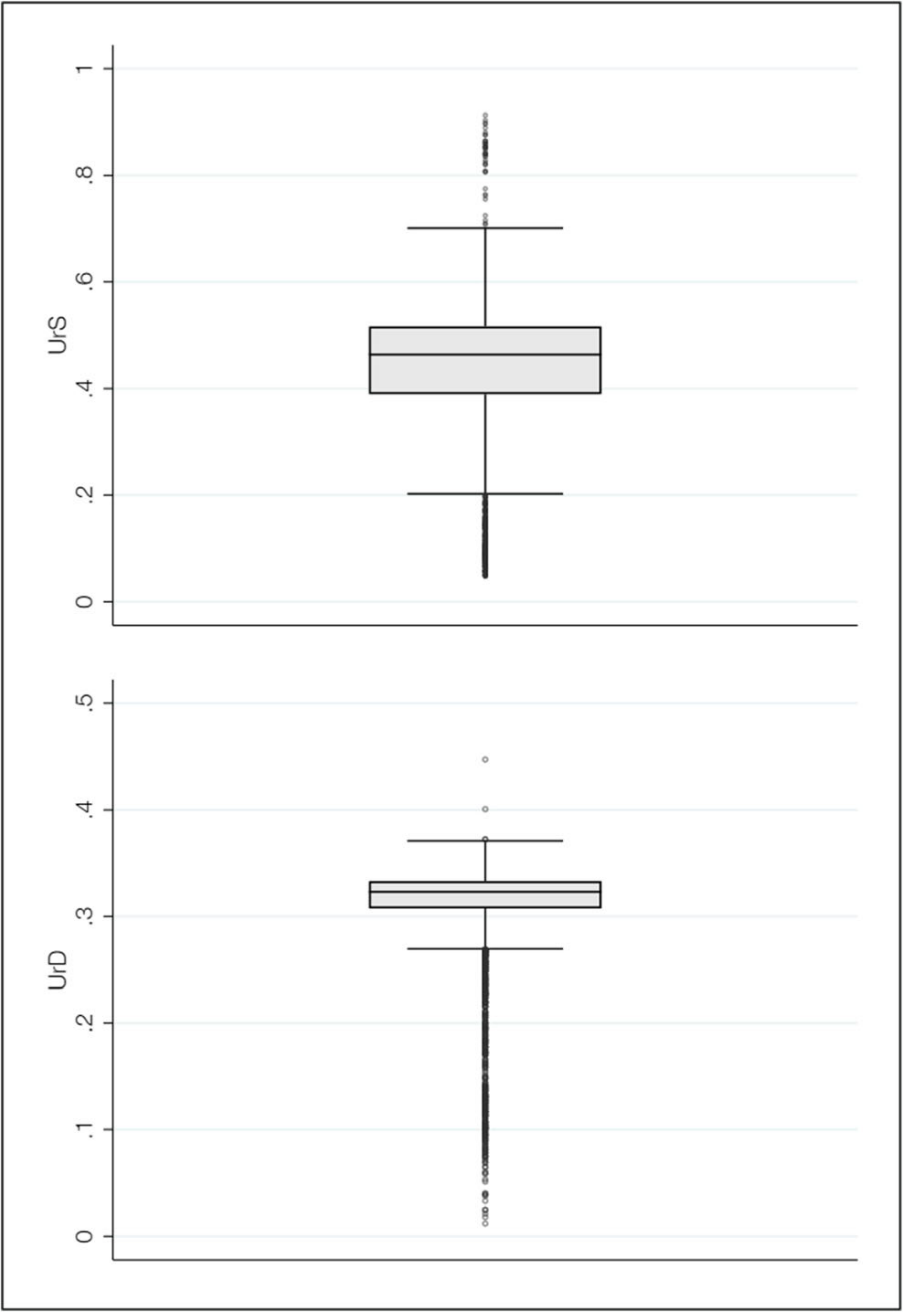
Boxplots of the average urgency score (UrS) and the within-hospital dispersion of medical urgency (UrD)

Table 2 presents measures and summary statistics of the inputs and outputs, as well as of the independent variables investigated in the double bootstrap truncated regression. The mean of the hospitals’ UrS was 0.44, which indicates that, on average, elective care predominated in the case composition of the hospitals in our sample. However, there was some variation across hospitals with respect to their UrS. As depicted in Fig. 1 (upper graph), our sample includes some hospitals with a case composition comprising mostly elective care (UrS < 0.5; 67.8%) and some with a case composition comprising mostly emergency care (UrS ≥ 0.5; 32.2%). The mean UrD in our sample was 0.31. The lower graph of Fig. 1 indicates that there were hospitals in which cases were relatively homogeneous in terms of their medical urgency, and hospitals in which the UrD was higher.

### Conventional and bias-corrected efficiency estimates

The results of the conventional and bias-corrected technical efficiency scores are presented in Table 3. To get a better understanding of the distribution of technical efficiency scores within our sample, we additionally calculated the share of hospitals that had a score of 1.00 (i.e., full technical efficiency), between 0.80–0.99, between 0.60–0.79, between 0.40–0.59, or below 0.40. The mean conventional technical efficiency score was 0.66, and the mean bias-corrected score was 0.55. The bias-corrected results indicate that, on average, hospitals could reduce their inputs by approximately 45%, while holding outputs constant, to reach full technical efficiency. When comparing the conventional and the bias-corrected technical efficiency scores, we found that the latter were lower, which suggests that conventional DEA estimates tend to be upward biased. Regarding the bias-corrected technical efficiency scores, none of the hospitals in our sample reached full efficiency. This is not surprising, however, given that the bias correction adjusts the estimated efficiency scores in such a way that they can never equal one [52]. In this context, Simar & Wilson [19] note that the relatively high share of fully efficient hospitals observed when using the conventional DEA approach may be induced by finite sample bias rather than by the true underlying data generating process. With bias-correction, the share of hospitals in the upper score ranges declines and the share of hospitals in the lower ranges increases.

**Table 3:** Conventional and bias-corrected technical efficiency scores

	Original TE	Bias-corrected TE
Mean	0.66	0.55
SD	0.16	0.13
Frequency of DMUs with TE score		
1.00	0.09	–
0.80–0.99	0.10	0.05
0.60–0.79	0.38	0.22
0.40–0.60	0.41	0.64
< 0.40	0.02	0.08

*n* = 4094; *DMU* decision making unit, *TE* technical efficiency

### Double bootstrap DEA results

**Fig. 2:**
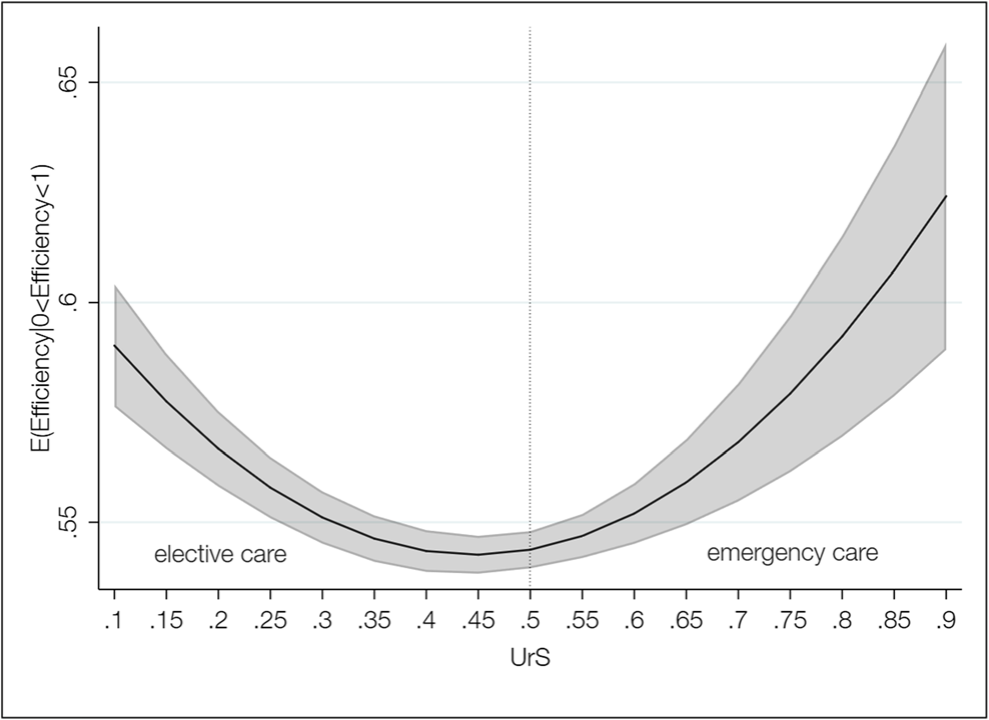
Predictive margins for different urgency scores (UrS) with 95% confidence intervals (Model II)

The parameter estimations obtained from the double bootstrap DEA with truncated regression are presented in Table 4. We built our models in blocks: First, we examined the linear effects of hospitals’ UrS (Model I). Second, to test for non-linear effects, we added a squared term for the UrS (Model II). Finally, we included the measure for UrD in a third model specification (Modell III). Regarding Model I, our results suggest that there was a significant negative relationship between hospitals’ UrS and technical efficiency. The squared UrS added in Model II was significant and positive, indicating a u-shaped relationship between hospitals’ UrS and technical efficiency.^7^ To analyze this relationship in more detail, we plotted predictive margins for different values of hospitals’ UrS, while keeping all other covariates constant (Fig. 2). Initially, the predicted level of technical efficiency decreased with increasing UrS until a turning point, i.e. between 0.4 and 0.5, after which technical efficiency increased again. We also observed that the 95% confidence intervals became larger in the higher ranges of the UrS, which is likely due to a lower number of observations in these ranges. Regarding the within-hospital UrD, our results suggest a significant negative association with hospital efficiency (Model III), indicating that the higher hospitals’ UrD the lower was their technical efficiency.

**Table 4:** Results from the truncated regressions with double bootstrap

Variable	Model I Coefficient (B-SE)	Modell II Coefficient (B-SE)	Model III Coefficient (B-SE)
UrS	−0.039 ** (0.016)	−0.356 *** (0.055)	
UrS^2^		0.399 *** (0.068)	
UrD			−0.259 *** (0.038)
Ownership – nonprofit^a^	−0.022 *** (0.005)	−0.022 *** (0.005)	−0.023 *** (0.005)
Ownership – for-profit^a^	0.013 ** (0.005)	0.009 * (0.006)	0.010 * (0.006)
Teaching status	−0.013 *** (0.004)	−0.010 ** (0.004)	−0.011 *** (0.004)
Competition (HHI)	0.119 *** (0.022)	0.126 *** (0.022)	0.125 *** (0.022)
Location - urban district^b^	0.018 *** (0.005)	0.019 *** (0.005)	0.019 *** (0.005)
Location - rural district^b^	0.008 (0.007)	0.008 (0.007)	0.007 (0.006)
Location - rural remote district^b^	−0.004 (0.008)	−0.004 (0.008)	−0.006 (0.007)
(Intercept)	0.558 *** (0.009)	0.612 *** (0.013)	0.621 *** (0.013)

*n* = 4094; bootstrapped standard errors (B-SE) in parentheses; year dummies (2016, 2017) included

*** significant at the 1% level, ** significant at the 5% level, * significant at the 10% level

^a^ reference group: public, ^b^ reference group: large cities

*HHI* Herfindahl- Hirschman Index, *UrD* within-hospital urgency dispersion, *UrS* urgency score

For control variables, our results indicate that, compared to public ownership, nonprofit ownership was negatively related to technical efficiency in all models. In contrast, we found that private for-profit hospitals operated at a significantly higher level of efficiency than public ones. Furthermore, our results suggest that academic teaching status was negatively associated with hospital efficiency in all models. With respect to the HHI, our results indicate that hospitals operating in less competitive markets seemed to be more efficient than hospitals operating in more competitive ones. For hospitals’ location, we found a positive and significant coefficient for urban districts, which suggests that hospitals located in densely populated districts operated more efficiently than hospitals in larger cities. The coefficients relating to the other locations, i.e., rural district and rural remote district, were not significant in either model specification. For all coefficients, we observed rather small effect sizes, albeit ones that were comparable to those found in other studies evaluating the determinants of hospital efficiency with the two-stage double bootstrap DEA approach [23, 31].

### Sensitivity analyses

To test the robustness of our results, we performed several sensitivity analyses. First, we excluded outpatient cases from our set of outputs because outpatient cases might differ with respect to case severity, which in turn could affect efficiency scores. So far, there is no reliable adjustment mechanism available to weight outpatient cases in a way similar to that done with inpatient adjustment [34]. Second, we re-ran our models with aggregated personnel inputs: a) we combined nursing FTEs, i.e. registered nurses and nurse assistants into one nursing FTE input, and b) we aggregated all FTE categories (physicians, registered nurses and assistant nurses) to one single FTE input using the aggregation procedure based on principal component analysis (PCA) suggested by Daraio and Simar [53]. Third, we used the approach of super-efficiency to detect DMUs that operated on a scale above the efficient frontier and thus to address concerns that our results might be affected by outlying observations. Following previous research, we therefore reduced our sample by excluding observations with (super-) efficiency scores, i.e. scores larger than 1.2 [54, 55], and re-ran the double-bootstrap truncated regression. Forth, we re-ran our regressions for individual observational years to ensure that our results were not affected by the sample size or serial correlation issues related to the pooled cross-sectional study design. Lastly, we re-estimated our models using an R software package (rDEA), which implements Simar and Wilsons’ algorithm #2 in terms of Shephard’s distance function. The results of all of these sensitivity analyses suggest that our findings were robust.^8^

## Discussion

The key innovation of our study was to explore the link between hospitals’ urgency characteristics and their technical efficiency by using (1) a measure of the average urgency in hospitals’ case composition, i.e. the hospitals’ UrS, and (2) a measure of how medical urgency is spread out across the hospitals’ case composition, i.e. the hospitals’ UrD. This multiple measure approach allows for the first time a detailed understanding of whether and how the urgency score and within-hospital urgency level dispersion affects hospitals’ efficiency.

Our results suggest that hospitals with higher UrS had lower technical efficiency (Model I). However, when we added a squared term of the UrS to test for non-linear effects, our results indicate that the relationship between a hospitals’ UrS and its efficiency was actually u-shaped (Model II). Given the predictive margins (Fig. 2), this finding suggests that an increase in the UrS among hospitals in which elective care predominates (i.e., with an overall UrS below 0.5) is detrimental for hospitals’ efficiency. In contrast, an increase in the UrS among hospitals in which emergency care predominates (i.e., with an UrS equal or above 0.5) is beneficial for technical efficiency. This suggests that how medical urgency is spread out across the hospitals’ case composition, i.e. the hospitals’ UrD, is crucial for understanding the urgency-efficiency link.

The results of Model III indicate that, as the UrD increases, a hospital’s technical efficiency decreases. In a hospital with a high UrD, processes may be more fragmented because urgent and less urgent cases both require the same resources, such as personnel or operating rooms [4, 14]. That is, the higher the fluctuation between urgent and less urgent cases, the greater is the risk for interruptions of routines, which ultimately might lead to efficiency losses at the organizational level. By contrast, a case composition that is homogeneous in terms of levels of medical urgency could allow hospitals to adapt their processes to the specific requirements related to the urgency of cases, which in turn might have beneficial effects on hospitals’ efficiency. Similar explanations can be found in the stream of literature linking the degree of specialization to hospitals’ efficiency. The findings of some of these studies suggest that a hospital’s specialization in specific diagnosis categories positively relates to their efficiency [17, 18]. In this context, it has often been argued that focusing on the treatment of similar hospital cases might lead to efficiency gains due to an enhancement of the expertise of staff through learning effects as well as to lower uncertainty surrounding the diagnosis and treatment of hospital cases within the specialization [43, 56]. The predictability of hospital demand is another factor to consider when examining how the UrD is related to hospitals’ efficiency. Prior research has found that higher demand uncertainty, defined by the difference between predicted and actual demand for emergency treatment, has detrimental effects on performance, because hospitals have to maintain resources that are ultimately used inefficiently or not at all [1, 16]. This line of reasoning might also be applicable to our findings in that hospitals facing demand uncertainty due to higher UrD might be struggling with resource optimization. In contrast, lower UrD might reduce demand uncertainty, which facilitates resource planning in a more efficient way.

Regarding the control variables, our results suggests that nonprofit hospitals are less technically efficient, and for-profit hospitals are more technically efficient, than public hospitals. So far, the evidence on the relationship between hospital ownership and hospital efficiency has been mixed [23, 41, 51, 57]. For instance, within the German hospital context, Tiemann and Schreyögg [51] also found nonprofit hospitals to be less efficient than public ones. However, in contrast to our results, they found private for-profit hospitals to be less efficient than public hospitals. One explanation could be that the target function of nonprofit hospitals is based more on charitable values than efficiency. Private hospitals, on the other hand, are more market- and profit-oriented, which encourages efficiency-like behavior. Regarding hospital teaching status, our results indicate that hospitals involved in academic teaching are less efficient than their non-teaching counterparts. This supports the general assumption that academic teaching activity has detrimental effects on hospital efficiency because it might reduce the productivity of medical labor [50]. With respect to teaching hospitals, other outputs such as teaching volume may be relevant which is not adequately captured by our production model. Furthermore, our study indicates that hospitals operating in less competitive markets tend to be more efficient than hospitals in markets that are highly competitive. This suggests that it might be easier for hospitals in less competitive markets to optimize resource use. This finding is in line with prior evidence on German hospitals [51]. Finally, our findings indicate that hospitals located in urban districts are more likely to operate efficiently than hospitals in large cities. This is in line with prior research showing a significant association between the geographical location and hospitals’ efficiency [32]. However, the coefficients for rural and remote locations were not significant in our model.

Although this study offers valuable novel insights into the link between medical urgency and hospital efficiency, its findings should be considered in light of two sets of limitations. The first set of limitations pertains to the methodological approach applied in this study. Using the two-stage approach to investigate variations in hospital efficiency, we assume that the ‘separability condition’ holds without explicitly testing this condition. We acknowledge that the threat of non-separability might still be an important limitation of our study. Disentangling the exact mechanisms through which hospitals’ urgency characteristics enter into the production process needs further investigation and is a promising avenue for future research. Another methodological limitation relates to the pooled cross-sectional study design. By pooling the data for the individual years, we indirectly assume that the production technology did not change during our study period. However, as we observe three consecutive years, i.e. a rather short time period, we argue that the risk of substantial changes in production technology is rather low. The second set of limitations pertains to the level of detail in our data set. For instance, we do not have individual patient-level data, which prevents us from using the DRG case mix index in adjusting the total number of inpatient cases. Further, our data does not contain information on costs and teaching outputs, which prevents us from analyzing additional inputs and outputs. Future research could extend our model by investigating additional inputs and outputs, such as further capital inputs (e.g., operational costs, expenses for technical equipment) or research and teaching outputs (e.g., number of peer-reviewed publications, teaching volume). Finally, future research might extend our model by empirically testing whether and how capacity constraints mediate the link between hospitals’ urgency characteristics and efficiency.

## Conclusion

One of the key finding of this study indicates that having a case composition, in which either elective care or emergency care predominates, positively influences hospitals’ efficiency. Furthermore, a case composition that is diverse in terms of levels of medical urgency negatively influences hospitals’ technical efficiency. From a management perspective, the findings point to the importance of considering the medical urgency of a hospital’s case composition when defining performance goals. Furthermore, focusing on similar urgent cases might be one means for hospitals to increase their efficiency. In addition to its practical relevance, this study has also some implications for further research. Future studies should consider urgency characteristics as important source for variations in hospitals’ efficiency. Another promising avenue for future research is to use qualitative process study approaches to closely investigate the mechanisms through which medical urgency cases lead to efficiency losses.

## Electronic supplementary material

ESM 1 (DOCX 61 kb)

## Footnotes

1 Even when reducing dimensionality to only one input and one output vector using the proposed dimension reduction method based on the first principal components, the results of our second-stage analyses remained stable. The results are available from the authors upon request.

2 We tested whether it is appropriate to assume VRS using the test statistics proposed by Simar and Wilson [27]. On a 1% significance level, we could reject the null hypotheses of CRS and therefore continued under the VRS assumption.

3 We acknowledge that proposing a radial model has the disadvantage that the potential of slack in technological constraints cannot be accounted for. An extension of our model by non-radial approaches, especially by incorporating input-slacks, e.g. through slacks-based models (SBM), would therefore be a promising avenue for further research applications. Another interesting extension of our model would be the incorporation of undesirable outputs, such as bad quality. Since integrating quality into nonparametric analysis of efficiency is not straightforward and different quality indicators have been shown to enter differently into the production process [35, 36], we did not include any quality parameters into our model, since the focus of our study is another.

4 In our specific study context, the case mix adjustment of inpatient cases is also important because hospitals with a high amount of urgent cases might also have a higher case mix index (implying longer LOS and resource use). Consequently, hospitals with higher UrS would score lower in efficiency.

5 The International Statistical Classification of Diseases and Related Health Problems, 10th Revision, German Modification (ICD-10-GM) is the official classification for coding diagnoses in outpatient and inpatient care in Germany. Although the classification scheme of Krämer et al. [3] was developed in the context of the German hospital market, the authors also provide urgency values for other ICD versions.

6 Please note that we regard hospitals’ case composition as being on a continuum between elective care and urgency care, rather than defining a clear cut off. Therefore, we include the UrS as continuous variable in our model.

7 To test whether Model II is statistically different from Model I, which is nested within Model II, we re-estimated these models using truncated regression analysis (without bootstrap) and bias-corrected efficiency scores as dependent variable. The Likelihood Ratio test statistics indicated that Model II fits our data significantly better than Model I. Therefore, we keep on interpreting Model II rather than Model I.

8 The results from the sensitivity analyses are available from the authors upon request.
