# Supplementary material for: Investigating the link between medical urgency and hospital efficiency – Insights from the German hospital market
Source: Health Care Manag Sci. 2020 Sep 16;23(4):649–60. doi: 10.1007/s10729-020-09520-6 (PMC7674330; doi:10.1007/s10729-020-09520-6)
Supplement: Supplementary file 1 — (DOCX 61 kb) [file 10729_2020_9520_MOESM1_ESM.docx]

**Electronic supplementary material**

**ESM 1:** Farrel input-orientated technical efficiency model under VRS

$\mathrm{Min}_{\lambda_{j}, \theta} \theta, subject to:$ (E1)

$$\sum_{j=1}^{n} \lambda_{j}x_{ij}\leq\theta x_{io} ; (i=1, \ldots, m)$$

$$\sum_{j=1}^{n} \lambda_{j}y_{rj} \geq y_{r0} ;(r=1, \ldots, s)$$

$$\sum_{j=1}^{n} \lambda_{j}=1$$

$$\lambda_{j} \geq0 ;(j=1, \ldots, n)$$

The optimization is made over the intensity parameter $\lambda_{j}$ and θ. The optimal value of $\theta$ ($\theta^{*}$), which is the technical efficiency score, cannot exceed 1. Further, $y_{rj}$ is a vector of *s* outputs produced and $x_{ij}$ a vector of *m* inputs used by hospital *j*.

**ESM 2:** Second-stage analysis

We assume that the true relationship between the hospitals’ efficiency and the contextual variables (defined in chapter 2.4) is given by

$\theta_{j}=\beta z_{j}+\varepsilon_{j}.$ (E2)

$\theta_{j}$ denotes the (true) technical efficiency score of hospital *j*, and $z_{j}$ is a vector of contextual factors that we assume to influence hospital efficiency with the corresponding estimated parameters *β*. $\varepsilon_{j}$ denotes the error term. Since the true efficiency score is unknown and must therefore be approximated using estimated DEA efficiency scores, we follow the algorithm #2 steps described by Simar and Wilson [19] (see ESM 3).

**ESM 3:** Steps of the double bootstrap procedure proposed by Simar and Wilson [19] (Algorithm #2)

b) Compute $\theta_{j}^{**}= z_{j}\hat{\hat{\beta}}+ {\tilde{\tilde{\varepsilon}}}_{j}$ for each $j=1, \ldots, n.$

c) Use truncated maximum likelihood estimation to regress$\theta_{j}^{**}$ on $z_{j}$ to obtain estimates of ${\hat{\hat{\beta}}}^{*}$and ${\hat{\hat{\sigma}}}^{*}$.

Use the bootstrap estimates in $\Delta$ obtained in step 6 and the original estimates of $\hat{\hat{\beta}}$ and $\hat{\hat{\sigma}}$ from step 5 to calculate the ($1-\alpha$) confidence interval for each element of $\beta$ and $\sigma$.

Obtain a set of bootstrap estimates by repeating the following steps (a-c)
$L_{2}$times.
${\Delta=\left\{ {\hat{\hat{\beta}}}_{jb}^{*} \right\}}_{b=1}^{L_{2}}$ $j=1, \ldots, n$

Use truncated maximum likelihood estimation to regress ${\hat{\hat{\theta}}}_{j}$ on $z_{j}$ to obtain estimates of $\hat{\hat{\beta}}$ and $\hat{\hat{\sigma}}$.

c) Generate pseudo data with $x_{j}^{*}=x_{j}\frac{\hat{\theta}_{j}}{\theta_{j}^{*}}$ and $y_{j}^{*}=y_{j}$; $j=1, \ldots, n$.

a) For each $j=1, \ldots, n$ draw $\varepsilon_{j}$ from a truncated normal distribution (N(0,${\hat{\hat{\sigma}}}^{2}$) with left truncation at ${-z}_{j}\hat{\hat{\beta}}$ and right truncation at $1{-z}_{j}\hat{\hat{\beta}}).$

6.) Second bootstrap iteration

3.) First bootstrap iteration

a) For each $j=1, \ldots, n$ draw $\varepsilon_{j}$ from a truncated normal distribution (N(0,$\hat{\sigma}^{2}$) with left truncation at ${-z}_{j}\hat{\beta}$ and right truncation at $1{-z}_{j}\hat{\beta}).$

b) Compute $\theta_{j}^{*}= z_{j}\hat{\beta}+ \tilde{\varepsilon}_{j}$ for each $j=1, \ldots, n.$

d) By using $x_{j}^{*}$ and $y_{j}^{*}$, now estimate $\hat{\theta}_{j}^{*}$ using (1); $j=1, \ldots, n.$

Obtain a set of bootstrap estimates by repeating the following steps (a-d) $L_{1}$ times.

${B_{j}=\left\{ \hat{\theta}_{jb}^{*} \right\}}_{b=1}^{L_{1}}$ $j=1, \ldots, n$

Use truncated maximum likelihood estimation to regress $\hat{\theta}_{j}$ on the set of explanatory variables $z_{j}$ to obtain estimates of coefficients $\hat{\beta}$ and standard errors $\hat{\sigma}$.

Use the original data to estimate the technical efficiency$\hat{\theta}_{j}$, using (E1)

1.) Calculate conventional DEA estimates

For each $j=1, \ldots, n$ calculate the bias corrected efficiency estimator ${\hat{\hat{\theta}}}_{j}$ by correcting the original DEA estimate $\hat{\theta}_{j}$obtained in step 1 by the estimate of bias obtained in step 3.

4.) Bias-corrected efficiency estimates

Calculate confidence intervals

2.) First (truncated) regression

5.) Second (truncated) regression
